# *Brain and Neuroscience Advances* – 2024 in review

**DOI:** 10.1177/23982128251317305

**Published:** 2025-02-06

**Authors:** Kate Baker

**Affiliations:** 1 MRC Cognition and Brain Sciences Unit, University of Cambridge, Cambridge, UK; 2 Department of Pathology, University of Cambridge, Cambridge, UK; 3 Department of Medical Genetics, University of Cambridge, Cambridge, UK

Journals, and science more broadly, are akin to a relay race. These are team enterprises, requiring talented and motivated individuals, dedicating their time to training over the long term, and then giving their maximal focus and effort to pursue the collective goal of a short, brilliant and memorable run together. Baton handovers are key! Although each race goes around the track, ending-up approximately back where you started, no race is ever the same, and incrementally the teams get stronger/smoother/faster so the entire field improves.

I’m no athlete, but 2024 has been a year of passing-on-the-baton at *Brain and Neuroscience Advances.* After a decade of developing our journal’s concept, spear-heading our launch and steering our development, Jeff Dalley handed on the Editor-in-Chief’s stick (and carrot) to me. We ran alongside each other for a year or so, and I’ve tried not to drop the journal’s ethos or processes now that I’m going it alone. In 2025, I will be joined on the track by a new EiC (watch this space), so that this relay team has fresh legs going forwards.

Our publication has burgeoned in 2024, and I’d like to highlight articles with a (loose) relay race theme:

- Original articles have passed on methodological batons – sharing experimental triumphs and tribulations so that neuroscience can move forward with greater reproducibility, interpretability and impact. For example, Ralph et al. (2024) highlighted the importance of both age and sex when interpreting rodent results, while McFall et al. (2024) shared their observation that housing conditions can have unexpected impacts on behavioural and physiological outcomes in the context of preclinical trials. Alladin et al. (2024a) highlighted the opportunities and challenges of investigating affective processes during childhood, using multiple physiological and behavioural methods – there is clear potential for neuroscience answers to neglected questions about emotional development and mental health in childhood.
- Registered reports (RRs) are a particularly powerful, sequential research process whereby expectations based on prior observations can be robustly tested. The RR from Serin et al. (2024) did not replicate previously observed theta-induced memory effects, calling into question models of multi-sensory binding within the hippocampus. Wilson et al. (2024) published a RR on reporting standards within systematic reviews (of animal models relating to neurodevelopmental disorders), highlighting the biases and inadequacies of the existing literature and providing a clear, evidence-based perspective on necessary future improvements.
- Review articles epitomise the community science element of our field, by gathering together many individual race reports to generate new perspectives and inform the evolution of neuroscientific knowledge and practice. This year we published reviews on time perception, impulsivity and ADHD by White and Dalley (2024), and the gut–brain axis of disgust by Alladin et al (2024b).
- Journal Clubs are a popular and effective mechanism of picking up the baton of a newly published article and carrying it forward. Journal Clubs communicate the context and key messages of a specialised article to the broad readership of *BNAdvances*. Recent Journal Club topics have encompassed multimodal visual neuroscience in rodents (Bailey et al., 2024), biomarker research in Alzheimer disease (Holt et al., 2024) and the contribution of thalamic GABA receptors to conscious awareness (Rawlings-Mortimer and Dalley, 2024).
- Meeting reports are a new feature of *BNAdvances* whereby the content and experience of a conference or symposium can be summarised for and shared with our readership. We were very proud to publish a report by Mitchell-Heggs and Tse (2024) of a Royal Society meeting celebrating the 50th anniversary of the discovery of Long-Term Potentiation, featuring interviews with the superstar relay team who established this fundamentally important field.
- And finally, we have launched BNA Insights, providing a new opportunity to stimulate debate and promote progress within neuroscience (perhaps akin to the commentary box for a broadcast athletics match). Our first BNA Insight by Ali et al. (2024) reflects on low confidence in neuroscience (neurophobia) expressed by students (medical students in particular), which will have significant impacts on future healthcare and research. Helpful strategies are proposed to tackle this challenge, and we hope to publish future evidence-based research to facilitate improvements in neuroscience education. In parallel, Logan et al. (2024) surveyed UK undergraduate neuroscience degrees, an important starting gun for evidence-based development of curricula and career development.

**Figure 1.**
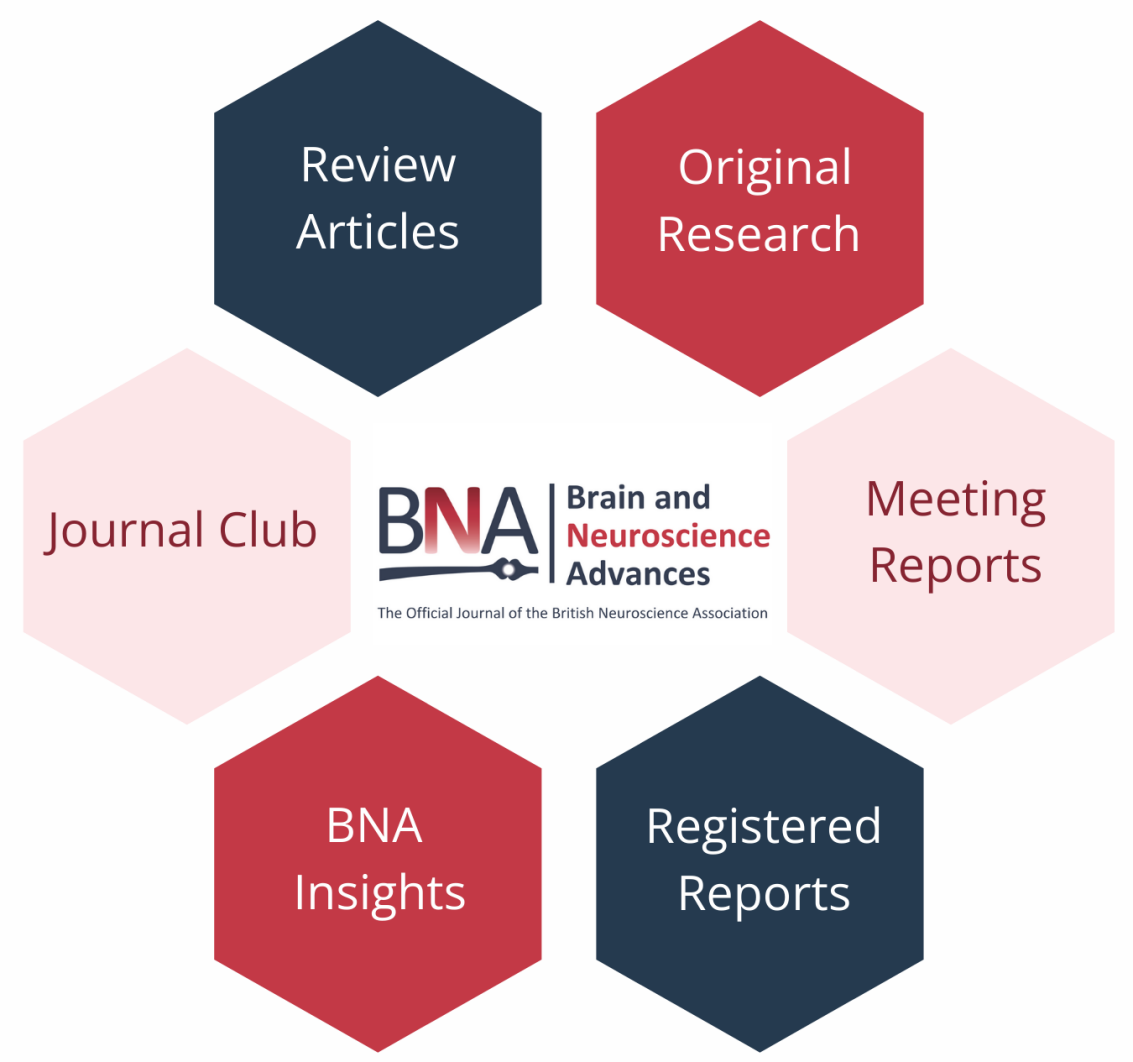
*Brain and Neuroscience Advances* article types 2025.

Summarising and reflecting, our journal’s strength this year has been the diversity of our articles (Figure 1). Few people would attend an athletics fixture if it only featured discus or 800 m (brilliant as those disciplines may be). It’s the variety of events, and the connections and contrasts between them, which probably makes the whole thing (neuroscience) fun!

My thanks to the many individuals who provided timely, high-quality peer reviews for submitted articles in 2024; to the BNA Chief Executive Laura Ajram and the BNA Council for their strategic support to the journal; and to our publishing team at Sage.

So what’s on the fixtures calendar for 2025? Based on my EiC inbox, I am confident that we will be publishing strong articles that consolidate and extend this diversity. *BNAdvances* is the society journal of the BNA, so excitement is mounting about the BNA*2025* International Festival of Neuroscience (https://meetings.bna.org.uk/bna2025/), where we will produce the second edition of Desert Island Papers and promote neuroscience writing as broadly as possible. I hope to see you there, and look forward to reading and publishing your *BNAdvances* submissions in 2025!
